# Effects of a 3-year dietary intervention on age-related changes in triglyceride and apolipoprotein A-V levels in patients with impaired fasting glucose or new-onset type 2 diabetes as a function of the APOA5 -1131 T > C polymorphism

**DOI:** 10.1186/1475-2891-13-40

**Published:** 2014-04-28

**Authors:** Minjoo Kim, Jey Sook Chae, Miri Kim, Sang-Hyun Lee, Jong Ho Lee

**Affiliations:** 1 National Leading Research Laboratory of Clinical Nutrigenetics/Nutrigenomics, Department of Food and Nutrition, College of Human Ecology, Yonsei University, 50 Yonsei-ro, Seodaemun-gu, Seoul 120-749, Korea; 2 Department of Food and Nutrition, Brain Korea 21 PLUS Project, College of Human Ecology, Yonsei University, Seoul, Korea; 3 Yonsei University Research Institute of Science for Aging, Yonsei University, Seoul, Korea; 4 Department of Family Practice, National Health Insurance Corporation Ilsan Hospital, Goyang, Korea

**Keywords:** Apolipoprotein A-V, *APOA5* -1131 T > C, Diabetes, Polymorphism

## Abstract

**Background:**

The purpose of this study was to estimate the effects of a 3-year dietary intervention on age-related changes in triglyceride and apolipoprotein (apo A-V) levels in patients with impaired fasting glucose (IFG) or new-onset type 2 diabetes as a function of the *APOA5* -1131 T > C polymorphism.

**Methods:**

We genotyped the *APOA5* -1131 T > C polymorphism in 203 Korean individuals with IFG or new-onset type 2 diabetes for the TT (n = 91), TC (n = 98), and CC (n = 14) alleles. Plasma apo A-V and triglyceride levels were evaluated at baseline and after a 3-year dietary intervention.

**Results:**

Our results showed that HDL, glucose, insulin, HOMA-IR index, free fatty acids, and apo A-V decreased and brachial-ankle pulse wave velocity (ba-PWV) and malondialdehyde (MDA) increased at the 3-year follow-up visit compared with baseline. Plasma apo A-V levels were reduced in subjects with the C allele (TC or CC) (P = 0.036) and triglyceride levels were reduced in subjects with the TT allele (*P* = 0.047). Subjects with the C allele showed lower post-treatment apo A-V and higher post-treatment fasting triglyceride levels than subjects with the TT allele. Changes in apo A-V and triglyceride levels were negatively correlated in subjects with the TT allele and positively correlated in subjects with the C allele.

**Conclusions:**

This study showed that the dietary intervention prevented an age-related increase in triglyceride levels in individuals with IFG or new-onset type 2 diabetes who possess the TT allele, but not the CT or CC allele, of the *APOA5* -1131 T > C polymorphism.

## Background

Apolipoprotein A-V (apo A-V), which was discovered in 2001, is part of the apolipoprotein family (APOA1/C3/A4). Apo A-V is encoded by the *APOA5* gene, which is located on human chromosome 11q23 and contains three introns and four exons [1-3]. Although apo A-V is mainly expressed in the liver in combination with the APO structure [2], apo A-V also exists in small amounts in the plasma and plays an important role in the regulation of triglyceride levels in both human and animal models. Pennacchio et al. found that triglyceride levels decreased approximately 66% in transgenic mice that overexpressed *APOA5* and were four times higher in *APOA5* knockout mice compared with control mice [1,3,4].

Elevated triglyceride levels are associated with abnormal lipid metabolism in patients with type 2 diabetes [1,5]. However, the mechanism underlying the modulation of triglyceride levels by plasma apo A-V as well as the effect of *APOA5* polymorphisms in humans has not been identified [4,6]. Many studies have shown that genetic variations in the *APOA5* gene are associated with hypertriglyceridemia and coronary heart disease [1,2,7]. However, few studies have explored genetic variations in the *APOA5* gene in individuals with type 2 diabetes. Therefore, we tested the effects of a 3-year dietary intervention on triglyceride and apo A-V levels in patients with impaired fasting glucose (IFG) or new-onset type 2 diabetes as a function of their *APOA5* polymorphisms.

The -1131 T > C single-nucleotide polymorphism (SNP) of the *APOA5* gene has a significant impact on diseases [5-7]. A previous study has shown that among healthy, non-obese Korean men who were genotyped with the TT, TC, or CC allele of the *APOA5* -1131 T > C polymorphism*,* triglyceride levels were higher in carriers of the C allele (TC or CC) compared with carriers of the TT allele. The levels of small dense low-density lipoprotein (LDL) cholesterol, high-sensitivity C-reactive protein, and lymphocyte DNA damage were also higher in carriers of the C allele compared with carriers of the TT allele. Therefore, the C allele of the *APOA5 -*1131 T > C polymorphism is significantly associated with risk factors for cardiovascular disease (CVD) [6]. Furthermore, Xu et al. performed a meta-analysis of the *APOA5 -*1131 T > C polymorphism among East Asian, European, and other ethnic populations and found that the C allele was associated with higher levels of fasting triglycerides, total cholesterol, and LDL cholesterol, and lower levels of high-density lipoprotein (HDL) cholesterol, especially in East Asian populations, in which metabolic syndrome was also increased by approximately 40% [8]. The C allele is more prevalent in Chinese (26-40%) than in Caucasian (8%) individuals [7]. Chandak et al. also found a higher prevalence of the C allele among Indians compared with Caucasians from the United Kingdom (20% vs. 4%, respectively; *P* < 0.001) [9]. Therefore, the C allele and disease-linked variants are common among the Asian population.

Carbohydrate-derived calories account for 65% of total caloric intake and refined rice is the primary source of carbohydrates among middle-aged adults, according the 2010 Korean National Health and Nutrition Examination Survey (KNHANES V-1). A high carbohydrate intake combined with a high prevalence of the *APOA5* -1131C allele may contribute to the relatively high mean serum triglyceride concentration (142 mg/dL) in the Korean population (KNHANES V-1). Previous observational studies and intervention trials have shown that the *APOA5* -1131 T > C polymorphism interacts with dietary factors, especially dietary fat, to determine triglyceride concentrations [10-14]. Lin et al. recently showed that elevated triglyceride levels were induced by a high carbohydrate diet in healthy individuals with the *APOA5* -1131C allele [15]. A clear association exists between diabetic dyslipidemia and the *APOA5* -1131 T > C polymorphism [16]. Furthermore, there is evidence that a high-legume, low glycemic index diet reduces fasting triglyceride levels in insulin-resistant subjects [17]. Therefore, we hypothesized that a 3-year dietary intervention with whole grains and legumes in a high-carbohydrate diet may mask the adverse effects of a high-carbohydrate intake among individuals with the C allele of the *APOA5* -1131 T > C polymorphism. Therefore, we studied the effects of a 3-year high-carbohydrate diet with whole grains and legumes on circulating levels of apo A-V and triglycerides in patients with IFG or new-onset type 2 diabetes as a function of the *APOA5* -1131 T > C polymorphism.

## Methods

### Study design and participants

Study subjects were recruited from the Health Service Center (HSC) during routine check-ups at the National Health Insurance Corporation Ilsan Hospital in Goyang, Korea between August 2006 and February 2008. Subjects were sedentary and had not participated in weight-reduction programs within the previous 3 years. Based on the data screened from the HSC, subjects with IFG (fasting glucose between 100 and 126 mg/dL) or new-onset type 2 diabetes (fasting glucose ≥126 mg/dL) were referred to the Department of Family Medicine or Internal Medicine. Their health and lipid profiles were re-screened, and subjects who satisfied the study criteria were recommended to participate in the dietary intervention program. Exclusion criteria included a current and/or past history of CVD, including angina, cancer, abnormal liver or renal function, thyroid or pituitary disease, and use of any medications (including anti-hypertensive, lipid lowering, anti-platelets, and anti-diabetic drugs). The purpose of the study was carefully explained to all potential subjects and their written consent was obtained prior to their participation. During the recruiting period, total 244 subjects were enrolled (TT: 109, TC: 118, CC: 17). Approximately 83% of subjects achieved, and finally, a total of 203 subjects completed the study. The study protocol was approved by the Institutional Review Board of Yonsei University and performed in accordance with the Declaration of Helsinki.

### Dietary intervention and assessment of dietary intake/physical activity level

Enrolled subjects visited the center one week before starting the intervention. Each subject’s habitual diet was obtained using both a 24-h recall method and a previously validated semi-quantitative food frequency questionnaire (SQFFQ) [18]. Analyses were conducted using the data from the 24-hour recall methods, and the SQFFQ was used to validate this data. All of the subjects were advised to continue their habitual diet for a week and were instructed to complete a 3-day food record (2 weekdays and 1 weekend day) as a baseline measurement. On the 3-day food record, subjects were instructed to weigh and record the amount of food before ingestion and any remaining food after ingestion. This 3-day record was compared with the 24-hour recall method to verify the habitual diet.

The dietary intervention program started one week after baseline measurements. The intervention program replaced each subject’s refined rice intake with equal parts of legumes, barley, and other whole grains three times per day as high-carbohydrate sources and increased their vegetable intake to at least 6 units (30–70 g/unit) per day to ensure a sufficient dietary fiber intake. To evaluate and reinforce compliance during the intervention period, dietitians interviewed the subjects bimonthly via telephone. Subjects were asked to report their 3-day food records at each visit. Subjects were also instructed to record their 24-hour physical activity in a diary every 4 weeks. Dietary energy values and nutrient content from the 3-day food records were calculated using the Computer-Aided Nutritional analysis program (CAN-pro 3.0, Korean Nutrition Society, Seoul, Korea). Total energy expenditure (kcal/day) was calculated from the 24-hour physical activity patterns [19], basal metabolic rate, and the specific dynamic actions of the food intake. Basal metabolic rate was calculated for each subject with the Harris–Benedict equation [20].

### Anthropometric parameters and blood collection

Body weight and height were measured to calculate body mass index (BMI, kg/m^2^). Waist circumference was measured at the umbilical level with the subject in the standing position after normal expiration and hip girth was measured at the widest part of the hip to calculate the waist to hip ratio (WHR). Blood pressure (BP) was measured in the left arm of seated patients with an automatic BP monitor (TM-2654, A&D, Tokyo, Japan) after 20 minutes of rest. Venous blood specimens were collected after a 12-hour fasting period in EDTA-treated and plain tubes that were centrifuged to produce either plasma or serum and stored at −70°C.

### Serum lipid profile, free fatty acids, fasting glucose, insulin concentration, and insulin resistance

Fasting serum concentrations of total cholesterol, triglycerides, and free fatty acids were measured using the Hitachi 7150 Autoanalyzer (Hitachi Ltd., Tokyo, Japan). Blood was precipitated using apoB-containing lipoproteins with dextran sulfate magnesium. Inter and intra-assay precision of total cholesterol was 1.73% and 1.52%, triglyceride was 2.52% and 1.28%, and free fatty acid was 3.73% and 0.99%, respectively. HDL concentrations in the supernatants were measured enzymatically. Inter and intra-assay precision was 1.61% and 1.37%. LDL was indirectly estimated in subjects with serum triglyceride concentrations less than 400 mg/dL using the Friedewald formula: LDL = total cholesterol - [HDL + (triglycerides/5)]. In subjects with serum triacylglycerol concentrations more than 400 mg/mL, LDL was measured directly using an enzymatic method on a Hitachi 7150 Autoanalyzer (Hitachi Ltd, Tokyo, Japan). Fasting glucose concentrations were measured with a glucose oxidase method from the Beckman Glucose Analyzer (Beckman Instruments, Irvine, CA). Inter and intra-assay precision was 1.14% and 0.94%. Insulin concentrations were measured by radioimmunoassay from the Immuno Nucleo Corporation (Stillwater, MN). Inter and intra-assay precision was 2.1% and 6.5%. The homeostasis model assessment of insulin resistance (HOMA-IR) was calculated using the following formula: HOMA-IR = [fasting insulin (μIU/mL) × fasting glucose (mmol/L)] × 22.5.

### Serum high-sensitivity C-reactive protein, plasma malondialdehyde, LDL particle size, and brachial-ankle pulse wave velocity

Serum high-sensitivity C-reactive protein (hs-CRP) concentrations were measured by the Express Plus™ auto-analyzer (Chiron Diagnostics Co., Walpole, MA, USA) and high-sensitivity CRP-Latex(II) *X*2 kit (Seiken Laboratories Ltd., Tokyo, Japan). Inter and intra-assay precision was 2.08% and 2.46%. Malondialdehyde (MDA) was measured by the TBARS Assay kit with thiobarbituric acid-reactive substances (Zepto-Metrix Co., Buffalo, NY, USA). Inter and intra-assay precision was 1.9% and 1.0%. Particle size distribution of LDL isolated by sequential flotation ultracentrifugation was examined by a pore gradient lipoprotein system (CBS Scientific, CA, USA) on commercially available, non-denaturing polyacrylamide slab gels containing a linear gradient of 2-16% acrylamide (Alamo Gels Inc., San Antonio, TX, USA). Standards of latex beads (34 nm) thyroglobulin (17 nm), apoferritin (12.2 nm), and catalase (10.4 nm) were used to estimate the relative migration rates of each band. Gels were scanned using a GS-800 Calibrated Imaging Densitometer (Bio-Rad, Graz, Austria). Inter and intra-assay precision was 2.29% and 3.88%. Brachial-ankle pulse wave velocity (ba-PWV) was measured by the automatic waveform analyzer (model VP-1000; Nippon Colin Ltd., Komaki, Japan). Subjects were examined in the supine position after 5 minutes of rest.

### Plasma apolipoprotein A-V and APOA5 -1131 T > C genotyping

Plasma apo A-V was measured by enzyme immunoassay (Human Apolipoprotein A ELISA kit, Millipore, MO). The results were read at 450 nm by the Victor2 (Perkin Elmer Life Sciences, Turka, Finland). Inter and intra-assay precision was 2.46% and 7.45%. Genomic DNA was extracted from 5 mL of whole blood using a commercially available DNA isolation kit (WIZARD® Genomic DNA purification kit, Promega Corp., Madison, WI, USA) according to the manufacturer’s instructions. Genotyping was performed by SNP-ITTM assays using single primer extension technology (SNPstream 25KTM System, Orchid BioSciences, NJ, USA). The colorimetric reactions were detected by an enzyme-linked immunosorbent assay (ELISA) reader, and the genotype was determined with QCReview™ software using methods that have been described previously [21].

### Statistical analysis

Statistical analyses were performed using SPSS version 12.0 for Windows (Statistical Package for the Social Sciences, SPSS Inc., Chicago, IL). The Hardy-Weinberg Equilibrium (HWE) analysis was performed using Haploview version 3.32 (http://www.broadinstitute.org/scientific-community/science/programs/medical-and-population-genetics/haploview/haploview). Prior to statistical analysis, each variable was tested for normality. Logarithmic transformations were performed on skewed variables. However, for graphical and interpretation purposes, values are reported as the untransformed variables. The effects of the dietary intervention were evaluated using a paired *t*-test. The genotype effects were tested by either a one-way analysis of variance (ANOVA) with a Bonferroni correction for multiple comparisons or an independent *t*-test. The association between changes in apo A-V concentrations and other metabolic variables was computed with Pearson and partial correlation coefficients. A general linear model (GLM) analysis was performed to compare confounding factors. Multiple regressions were performed to determine the independent predictors of apo A-V changes. The results are reported as the mean ± SE. *P* < 0.05 was considered statistically significant.

## Results

### Distribution of the APOA5 -1131 T > C polymorphism

Among 203 Korean patients with IFG or new-onset type 2 diabetes, 91 were homozygous (TT) for the T allele, 98 were heterozygous for the C allele (TC), and 14 were homozygous (CC) for the C allele of the *APOA5* -1131 T > C polymorphism. These frequencies did not deviate significantly from the Hardy-Weinberg equilibrium. The frequency of the C allele was 0.310, which is slightly higher than the frequency that was previously reported among a healthy Korean population (0.285) [22]. To increase statistical power, we pooled carriers of the less common allele (TC and CC).

### Clinical characteristics and nutrient intake before and after a 3-year dietary intervention

At the 3-year follow-up visit, fasting HDL, glucose, insulin, HOMA-IR, free fatty acids, and apo A-V had decreased, and MDA and ba-PWV had increased from baseline across all subjects (Table 1). There were significant increases from baseline in both fiber intake and the ratio of polyunsaturated to saturated fatty acids as well as a slight decrease in total energy expenditure and total energy intake. The mean BMI of the subjects did not change, and no significant change was observed in other biochemical markers (Table 1).

**Table 1:** Effects of a 3-year dietary intervention on anthropometric and biochemical markers

	**Pre-treatment**	**Post-treatment**	***P*-value**
Male/female (%)	58.6/41.4		
Age (year)	48.2±0.64	51.3±0.64	<0.001
Weight (kg)	66.6±0.72	66.3±0.71	0.210
Body mass index (kg/m^2^)	24.6±0.21	24.5±0.20	0.285
Systolic BP (mmHg)	121.1±0.97	122.4±0.96	0.158
Diastolic BP (mmHg)	74.9±0.72	75.8±0.75	0.171
Total cholesterol (mg/dL)^∮^	194.8±2.29	198.0±2.87	0.362
LDL cholesterol (mg/dL)^∮^	117.9±2.20	123.2±2.73	0.110
HDL cholesterol (mg/dL)^∮^	52.7±0.98	50.3±1.02	0.002
Triglycerides (mg/dL)^∮^	122.8±4.93	127.6±5.82	0.714
Glucose (mg/dL)^∮^	109.8±0.67	108.3±1.28	0.004
Insulin (μIU/mL)^∮^	9.04±0.26	8.27±0.27	<0.001
^1^HOMA-IR^∮^	2.47±0.08	2.24±0.09	<0.001
^2^hs-CRP (mg/dL)^∮^	1.24±0.12	1.31±0.27	0.451
Free fatty acids (μEq/L)^∮^	531.4±15.1	498.7±16.2	0.014
Apolipoprotein A-V (ng/mL)^∮^	202.4±7.44	186.4±6.85	0.030
^3^ba-PWV (cm/sec)^∮^	2694.2±28.7	2758.3±31.1	0.002
^4^MDA (nmol/mL)^∮^	10.4±0.21	11.4±0.27	0.003
Total energy expenditure (kcal)	2146.8±21.5	2118.5±18.4	0.012
Estimates of daily nutrient intake			
Energy intake (kcal)	2196.3±22.3	2162.7±20.3	0.009
Carbohydrate (%)	61.7±0.10	57.5±0.07	<0.001
Protein (%)	16.9±0.08	17.5±0.06	<0.001
Fat (%)	21.7±0.11	22.9±0.09	<0.001
Crude fiber (g)^∮^	11.3±0.43	15.0±0.56	<0.001
^5^PUFA/SFA^∮^	1.73±0.07	2.08±0.08	0.003

Mean ± SE, n = 203. ^∮^Log-transformed, *P*-values derived from paired *t*-test.

^1^HOMA-IR = {fasting insulin (μIU/mL) × fasting glucose (mmol/L)}/22.5. ^2^hs-CRP = high sensitivity C-reactive protein. ^3^ba-PWV = brachial-ankle pulse wave velocity. ^4^MDA = malondialdehyde. ^5^PUFA/SFA = ratio of polyunsaturated to saturated fatty acids.

### Effects of a 3-year dietary intervention as a function of the APOA5 -1131 T > C genotype

**Figure 1:**
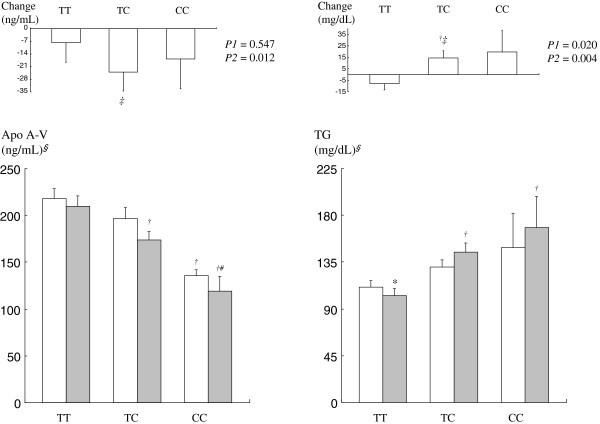
**Fasting apolipoprotein A-V and triglyceride levels at baseline (white square) and the 3-year follow-up visit (gray square) and mean changes as a function of the *APOA5 *-1131 T > C genotype.** Data (mean ± SE) included 91 (TT), 98 (TC), and 14 (CC) subjects. ^*§*^Log-transformed. *P1*, unadjusted *P* value; *P2*, baseline-adjusted *P* value. ^*^P < 0.05 compared with baseline values for each genotype by paired *t*-test. ^*†*^*P* < 0.05 compared with the TT allele; ^*‡*^*P* < 0.05 compared with the TT allele after adjusting for baseline values; ^*#*^*P* < 0.05 compared with the TC allele tested by a one-way ANOVA with a Bonferroni correction.

There were no significant differences in the distribution of sex, age, BMI, WHR, BP, total cholesterol, LDL, hs-CRP, or estimates of daily nutrient intake across the *APOA5* -1131 T > C genotypes at baseline or the 3-year follow-up visit (data not shown). The mean (±SEs) fasting apo A-V and triglyceride levels at baseline and the 3-year follow-up visit across the *APOA5* -1131 T > C genotypes are shown in Figure 1. At baseline, there was a significant association between apo A-V levels and the -1131 T > C genotype (*P* = 0.009) such that apo A-V levels were approximately 10% and 38% lower in TC and CC allele carriers, respectively, compared with TT allele carriers. Triglyceride levels were also approximately 17% and 34% higher in TC and CC allele carriers, respectively, compared with TT allele carriers at baseline. At the 3-year follow-up visit, plasma apo A-V levels were reduced in C allele carriers (*P* = 0.036) and fasting triglyceride levels were reduced in TT allele carriers (*P* = 0.047) (Table 2). After adjusting for baseline levels, significant associations were found between apo A-V changes and the *APOA5* genotype (TT: −7.77 ± 10.79 ng/mL; TC: −23.99 ± 10.52; CC: −16.71 ± 16.11; *P* = 0.012) and between triglyceride changes and the *APOA5* genotype (TT: −7.88 ± 5.44 mg/dL; TC: 14.38 ± 6.21; CC: 19.57 ± 18.58; *P* = 0.004) (Figure 1). TC or CC allele carriers had significantly lower post-treatment apo A-V levels and higher post-treatment fasting triglyceride levels than TT allele carriers.

**Table 2:** **Effects of a 3-year dietary intervention on apolipoprotein A-V, triglycerides, HDL, glucose, insulin, HOMA-IR, LDL particle size, MDA, and ba-PWV at baseline and the 3-year follow-up visit as a function of the *APOA5 *-1131 T > C genotype**

	***APOA5 *-1131 T > C**		***P*-value**	***P***^***a***^**-value**
	**TT (n = 91)**	**TC and CC (n = 112)**		
Apolipoprotein A-V (ng/mL)				
Before^*∮*^	217.8±10.6	188.6±10.2	0.011	
After^∮^	210.0±10.8	166.4±8.32^*^	0.001	
Change	−7.77±10.8	−23.0±9.35	0.285	0.004
Triglycerides (mg/dL)				
Before^∮^	110.9±6.20	132.5±7.28	0.022	
After^∮^	103.0±6.88^*^	147.6±8.51	<0.001	
Change	−7.88±5.44	15.0±5.88	0.005	0.001
HDL cholesterol (mg/dL)				
Before^∮^	55.1±1.53	50.8±1.26	0.024	
After^∮^	53.5±1.46	47.7±1.39^**^	0.001	
Change	−1.62±1.15	−3.11±1.18	0.372	0.051
Glucose (mg/dL)				
Before^∮^	109.7±1.00	109.9±0.90	0.874	
After^∮^	105.7±1.10^***^	110.4±2.11	0.064	
Change	−4.07±0.98	0.46±1.68	0.030	0.030
Insulin (μIU/mL)				
Before^∮^	9.19±0.36	8.93±0.37	0.425	
After^∮^	7.96±0.36^***^	8.52±0.39	0.375	
Change	−1.23±0.38	−0.41±0.35	0.115	0.173
^1^HOMA-IR				
Before^∮^	2.51±0.11	2.44±0.11	0.469	
After^∮^	2.10±0.10^***^	2.36±0.13	0.177	
Change	−0.39±0.11	−0.07±0.10	0.034	0.047
Free fatty acids (μEq/L)				
Before^∮^	540.8±22.8	523.7±20.3	0.577	
After^∮^	494.2±24.1^*^	502.4±22.0	0.836	
Change	−46.6±25.6	−21.3±24.9	0.481	0.620
LDL particle size (nm)				
Before^∮^	23.7±0.10	23.7±0.08	0.648	
After^∮^	23.8±0.11	23.4±0.09^*^	0.008	
Change	0.10±0.09	−0.23±0.09	0.014	0.006
^2^MDA (nmol/mL)				
Before^∮^	10.1±0.31	10.5±0.29	0.418	
After^∮^	10.7±0.42	11.9±0.35^***^	0.020	
Change	0.48±0.39	1.38±0.32	0.078	0.048
^3^ba-PWV (cm/sec)				
Before^∮^	2678.0±36.4	2707.3±42.9	0.736	
After^∮^	2701.4±38.9	2804.6±46.4^***^	0.134	
Change	23.4±29.7	97.3±25.3	0.058	0.046

Mean ± S.E.^∮^Log-transformed, ^*^P < 0.05,^**^P < 0.01,^***^P < 0.001 compared with baseline values for each genotype tested by paired *t*-test. *P*-values derived from independent *t*-test. *P*^*a*^-values derived from independent *t*-test after adjusting for baseline values. ^1^HOMA-IR = {fasting insulin (μIU/mL) × fasting glucose (mmol/L)}/22.5. ^2^MDA = malondialdehyde. ^3^ba-PWV = brachial-ankle pulse wave velocity.

The mean (±SEs) fasting HDL, glucose, insulin, HOMA-IR index, free fatty acids, LDL particle size, MDA, and ba-PWV values at both baseline and the 3-year follow-up visit are reported in Table 2 as a function of the *APOA5* -1131 T > C genotype. At baseline, C allele carriers had lower HDL levels (*P* = 0.024) than TT allele carriers. At the 3-year follow-up visit, fasting glucose (*P* < 0.001), insulin (*P* < 0.001), the HOMA-IR index (*P* < 0.001), and free fatty acids (*P* = 0.020) were reduced in TT allele carriers but not C allele carriers. C allele carriers had decreased HDL levels (*P* = 0.005) and LDL particle size (*P* = 0.048) and increased MDA (*P* < 0.001) and ba-PWV (*P* < 0.001) at the 3-year follow-up visit compared with baseline.

TT allele carriers had a greater reduction in glucose (*P* = 0.030) and the HOMA-IR index (*P* = 0.047) than C allele carriers after adjusting for baseline levels. C allele carriers had a greater reduction in LDL particle size (*P* = 0.006) and a greater increase in plasma MDA (*P* = 0.048) and ba-PWV (*P* = 0.046) than TT allele carriers after adjusting for baseline levels. C allele carriers also had lower post-treatment HDL levels (*P* = 0.001), smaller post-treatment LDL particle sizes (*P* = 0.008), and higher post-treatment MDA (*P* = 0.020) than TT allele carriers (Table 2).

### Relationship between changes in plasma apo A-V levels and metabolic parameters

Correlations between the changes in apo A-V levels and metabolic parameters were determined after adjusting for age, sex, and changes in body weight (*r*_*1*_), initial triglyceride levels (*r*_*2*_), and initial apo A-V levels (*r*_*3*_) (Table 3). Across all subjects, changes in apo A-V levels were negatively correlated with baseline apo A-V levels (*r*_*1*_ = −0.543, *r*_*2*_ = −0.555, all *P* < 0.001). In TT allele carriers, changes in apo A-V levels were negatively correlated with baseline apo A-V levels (*r*_*1*_ = −0.535, *r*_*2*_ = −0.552, all *p* < 0.001) and changes in triglyceride levels (*r*_*3*_ = −0.299, *p*_*3*_ = 0.006). In C allele carriers, changes in apo A-V levels were negatively correlated with baseline apo A-V levels (*r*_*1*_ = −0.613, *r*_*2*_ = −0.617, all of *P* < 0.001), but positively correlated with changes in triglyceride levels (*r*_*1*_ = 0.207, *p*_*1*_ = 0.038; *r*_*2*_ = 0.221, *p*_*2*_ = 0.028).

**Table 3:** Relationship between changes in plasma apolipoprotein A-V levels and metabolic parameters

	**Changed apo A-V levels**																	
	**All subjects**						**TT allele**						**C allele (TC and CC)**					
	***r***_***1***_	***p***_***1***_	***r***_***2***_	***p***_***2***_	***r***_***3***_	***p***_***3***_	***r***_***1***_	***p***_***1***_	***r***_***2***_	***p***_***2***_	***r***_***3***_	***p***_***3***_	***r***_***1***_	***p***_***1***_	***r***_***2***_	***p***_***2***_	***r***_***3***_	***p***_***3***_
Baseline levels																		
Triglyceride^∮^	−0.018	0.809	-	-	-	-	−0.082	0.456	-	-	-	-	0.052	0.608	-	-	-	-
Apo A-V^∮^	−0.543	<0.001	−0.555	<0.001	-	-	−0.535	<0.001	−0.552	<0.001	-	-	−0.613	<0.001	−0.617	<0.001	-	-
Free fatty acids^∮^	−0.159	0.030	−0.158	0.031	−0.047	0.529	−0.086	0.439	−0.066	0.554	−0.050	0.656	−0.237	0.017	−0.233	0.020	−0.036	0.722
HDL cholesterol^∮^	−0.203	0.005	−0.221	0.002	−0.105	0.153	−0.156	0.156	−0.173	0.118	−0.111	0.323	−0.263	0.008	−0.266	0.008	−0.107	0.295
Changed levels																		
Free fatty acids	0.275	<0.001	0.275	<0.001	0.247	0.001	0.231	0.035	0.226	0.040	0.292	0.008	0.304	0.002	0.301	0.002	0.190	0.060
Triglycerides	0.022	0.767	0.019	0.800	−0.084	0.257	−0.166	0.131	−0.195	0.077	−0.299	0.006	0.207	0.038	0.221	0.028	0.151	0.138
HDL cholesterol	0.366	<0.001	0.365	<0.001	0.354	<0.001	0.411	<0.001	0.405	<0.001	0.412	<0.001	0.313	0.002	0.310	0.002	0.266	0.008

^∮^Log-transformed.

Tested by Partial correlation analysis.

*r*_*1*_: correlation coefficient after adjusting for age, sex, and changes in body weight.

*r*_*2*_: correlation coefficient after adjusting for age, sex, changes in body weight and initial triglyceride levels.

*r*_*3*_: correlation coefficient after adjusting for age, sex, changes in body weight, initial triglyceride levels, and initial apo A-V levels.

### Independent predictors of apo A-V and triglyceride changes

Based on these results, we performed a multiple regression to determine independent predictors of apo A-V changes (Table 4). The *APOA5* -1131 T > C genotype, age, baseline BMI, baseline apo A-V levels, baseline triglyceride levels, change in triglyceride levels, baseline HDL, change in HDL, baseline glucose, change in glucose, baseline HOMA-IR, change in HOMA-IR, baseline free fatty acids, and change in free fatty acids were tested. The *APOA5* -1131 T > C genotype emerged as an independent predictor of changes in apo A-V levels (β = −24.532 ± 11.686, *P* = 0.037) along with baseline apo A-V levels (β = −107.870 ± 12.445, *P* < 0.001) across all subjects. Baseline apo A-V levels and changes in triglyceride levels and changes in HDL cholesterol levels also emerged as independent predictors of changes in apo A-V levels (β = −112.767 ± 19.550, *P* < 0.001; β = −0.404 ± 0.191, *P* = 0.038; β = 3.280 ± 1.007, *P* = 0.002, respectively) in both TT and C allele carriers (β = −101.958 ± 16.096; *P* < 0.001, β = 0.349 ± 0.133, *P* = 0.010; β = 2.239 ± 0.666, *P* = 0.001, respectively). To figure out the independent predictors of triglyceride changes, *APOA5* -1131 T > C genotype, age, baseline BMI, baseline apo A-V levels, baseline triglyceride levels, change in triglyceride levels, change in HDL, baseline glucose, change in glucose, baseline HOMA-IR, and change in HOMA-IR were tested (data not shown). The *APOA5* -1131 T > C genotype emerged as an independent predictor of changes in triglyceride levels (β = 17.668 ± 8.200, *P* = 0.033) along with baseline triglyceride levels (β = −35.455 ± 8.401, *P* < 0.001) and baseline apo A-V levels (β = −20.856 ± 10.025, *P* = 0.039) across all subjects. Baseline triglyceride levels and changes in apo A-V levels also emerged as independent predictors of changes in triglyceride levels (β = −36.641 ± 11.743, *P* = 0.003 and β = −0.179 ± 0.068, *P* = 0.010, respectively) in both TT and C allele carriers (β = −29.611 ± 12.206, *P* = 0.017 and β = 0.182 ± 0.078, *P* = 0.001, respectively). However, baseline apo A-V levels had shown as predictors of changes in triglyceride levels only in TT allele (β = −31.465 ± 13.668, *P* = 0.024).

**Table 4:** Multiple regressions to assess independent relationships between apolipoprotein A-V and clinical variables

**Variable**	**Changed apo A-V levels**					
	**Total subjects**		**TT allele**		**C allele (TC and CC)**	
	**Regression coefficients**	***P*-value**	**Regression coefficients**	***P*-value**	**Regression coefficients**	***P*-value**
	**[95% CI**^**†**^**]**		**[95% CI**^**†**^**]**		**[95% CI**^**†**^**]**	
*APOA5* -1131 T > C genotype	−24.532 [−47.596, −1.469]	0.037	-	-	-	-
Age	0.427 [−0.839, 1.693]	0.507	0.477 [−1.512, 2.465]	0.634	−0.204 [−1.880, 1.471]	0.809
Baseline levels						
Body mass index	1.078 [−3.811, 5.966]	0.664	0.812 [−6.501, 8.124]	0.826	2.696 [−4.004, 9.396]	0.426
Apo A-V^∮^	−107.870 [−132.432, −83.308]	<0.001	−112.767 [−151.730, −73.804]	<0.001	−101.958 [−133.939, −69.976]	<0.001
Triglycerides^∮^	−17.959 [−43.321, 7.402]	0.164	−19.362 [−61.602, 22.877]	0.364	−14.284 [−48.109, 19.542]	0.404
HDL cholesterol^∮^	24.125 [−31.326, 79.576]	0.392	−2.779 [−88.093, 82.536]	0.948	49.796 [−27.506, 127.097]	0.204
Glucose^∮^	−33.234 [−182.983, 116.516]	0.662	−210.610 [−463.912, 42.691]	0.102	129.957 [−64.812, 324.727]	0.188
HOMA-IR^∮^	22.975 [−12.858, 58.809]	0.207	12.221 [−49.304, 73.746]	0.693	14.817 [−30.146, 59.781]	0.514
Free fatty acids^∮^	26.503 [−4.251, 57.257]	0.551	42.963 [−5.154, 91.081]	0.079	9.158 [−32.164, 50.480]	0.661
Changed levels						
Triglycerides^∮^	0.066 [−0.152, 0.284]	0.551	−0.404 [−0.784, −0.024]	0.038	0.349 [0.085, 0.612]	0.010
HDL cholesterol^∮^	2.550 [1.455, 3.645]	<0.001	3.280 [1.274, 5.287]	0.002	2.239 [0.915, 3.563]	0.001
Glucose^∮^	−0.121 [−1.018, 0.775]	0.790	−0.631 [−2.905, 1.643]	0.582	−0.363 [−1.397, 0.671]	0.487
HOMA-IR^∮^	−4.036 [−17.793, 9.721]	0.563	0.119 [−22.914, 23.152]	0.992	−8.468 [−25.418, 8.481]	0.324
Free fatty acids^∮^	0.085 [0.034, 0.135]	0.001	0.079 [−0.005, 0.164]	0.065	0.071 [0.006, 0.137]	0.034
	*R*^2^ = 0.459, adjusted *R*^2^ = 0.416		*R*^2^ = 0.524, adjusted *R*^2^ = 0.440		*R*^2^ = 0.525, adjusted *R*^2^ = 0.455	
	*P* < 0.001		*P* < 0.001		*P* < 0.001	

^∮^Log-transformed. ^†^Confidence interval.

## Discussion

This study shows an effect of the *APOA5* -1131 T > C polymorphism in patients with IFG or type 2 diabetes among the Korean population. The frequencies of the *APOA5* -1131 T > C alleles (TT: 44.8%, TC: 48.3%, CC: 6.9%) did not deviate from the Hardy-Weinberg equilibrium. Our data showed that the TT allele is associated with lower triglyceride levels compared with the C allele, which agrees with a study by Xu et al. that demonstrated higher levels of fasting triglycerides, total cholesterol, LDL, and HDL, as well as a 33% increased risk of metabolic syndrome, in 51,868 Chinese subjects with the C allele [8]. In our previous research, CC allele carriers showed lower apo A-V levels than TT allele carriers, and showed association with greater triglyceride levels, smaller LDL particle size, and lower HDL-cholesterol [23]. Similarly, Li et al. found a higher C allele frequency among individuals with high total cholesterol compared with individuals with normal total cholesterol levels in both ethnic populations (Hei Yi Zhuang and Han Chinese; *P* < 0.05) [24]. However, a 15-year follow-up study of healthy men in the United Kingdom (the Northwick Park Heart Study II) showed no effects of the *APOA5* -1131 T > C polymorphism on the risk of type 2 diabetes [25]. This discordance across studies may be related to the differences among ethnic groups. The C allele of the *APOA5* -1131 T > C is more common among Asian populations (34% frequency in Japanese, 28-30% in Koreans, and 7.4% in Caucasians) [26,27]. The living conditions and progression of the disease as well as other subject characteristics may also affect the results.

The number of people diagnosed with diabetes worldwide is approximately 171 million, with a predicted increase to 366 million people by 2030 [28]. Elevated triglyceride levels as well as changes in plasma lipoproteins are associated with diabetes [24]. In the Framingham Heart Study, high VLDL levels which prompted the consideration of high triglyceride levels were associated with the development of diabetes, especially among subjects who were obese [29]. The relationship between obesity and diabetes is interdependent. Therefore, diet and exercise are extremely important for patients with diabetes.

Whole grain and legume consumption is known to decrease insulin resistance or insulin demand and improve lipid profiles, including decreasing triglyceride levels [30]. In particular, soy intake has been associated with increased HDL and decreased triglyceride levels [31]. This association is consistent with our finding that TT allele carriers who consumed whole grains and legumes had a significant decrease in fasting triglyceride levels. In contrast, C allele carriers had an increase in fasting triglyceride levels despite adhering to the same diet. This observation suggests that the magnitude of the effect of the *APOA5* gene on triglyceride levels is masked when metabolic conditions improved as a consequence of the whole grain and legume diet. This result is consistent with previous reports that lifestyle modification attenuates the biological effect of a genetic predisposition on metabolic profiles and diabetes risk [32].

Exercise has also been shown to increase insulin sensitivity in patients with type 2 diabetes [33]. Another study showed that exercise increased insulin sensitivity in both type 2 diabetes and healthy control subjects with the same age, BMI, and initial triglyceride levels. However, although exercise had little effect on triglyceride levels in control subjects, exercise significantly decreased triglyceride levels in patients with type 2 diabetes. Therefore, exercise may be therapeutic for abnormal lipid metabolism [34]. This result suggests that if exercise is therapeutic only for abnormal lipid metabolism, then an innate human genome sequence for this effect may not exist. Previous research has suggested that the promoter variant -1131 T > C is in strong linkage disequilibrium with *APOA5* -3A > G, which occurs in the ‘Kozak’ sequence in *APOA5*, and disruption of this sequence has been shown in other genes to severely disrupt ribosomal translation efficiency, leading to lower levels of apo A-V from this mRNA. Therefore, the C allele may itself not be functional, but is acting as a marker for the -3A > G that may contribute to lower apo A-V levels [35]. More studies are needed to determine whether diet and exercise can improve apo A-V levels in individuals with the C allele.

Apo A-V would increase the binding of TG-rich lipoproteins to glycosaminoglycans present on the surface of endothelial cells, thus rendering these lipoproteins more accessible to LPL [36]. Therefore, decreased apo A-V levels may reduce LPL-mediated TG hydrolysis of chylomicrons and VLDL and delay the clearance of lipoprotein remnants by the liver [37]. After hydrolysis of TG-rich lipoproteins by LPL, surface components are detached to form native HDL of discoidal form [38]. Thus, the delayed TG hydrolysis, attributable to apo A-V deficiency, reduces the availability of surface components of triglyceride-rich lipoproteins, thereby leading to a decreased formation of HDL-cholesterol. In this way, apo A-V is associated with HDL formation, and this may result in significant contribution of HDL as independent predictors for changes in apo A-V levels.

There are several limitations in our study. First, dietary intake during the 3-year dietary intervention was based on self-reports obtained from weighed food. However, measurement errors from self-reported dietary intake and lifestyle variables are relatively small [39]. Second, the euglycemic glucose clamp technique and 2-hour glucose tolerance test were not performed. Finally, we focused on individuals with IFG or new-onset type 2 diabetes; therefore, our results cannot be generalized to the greater population. Despite these limitations, we found that the *APOA5* -1131 T > C polymorphism plays an important role in the metabolic response to a 3-year dietary intervention in patients with IFG or new-onset type 2 diabetes. Whole-grain ingestion prevented the age-related increase in triglyceride levels in patients with IFG or new-onset type 2 diabetes who carried the TT allele but not the C allele of the *APOA5* -1131 T > C polymorphism. Our results suggest that the C allele contributes to the increased triglyceride levels among the Korean population. In addition, a positive relationship between triglyceride levels and the C allele may affect the onset of diabetes. However, long-term case–control studies and studies of the underlying mechanisms are needed to determine the relationship between the *APOA5* -1131 T > C polymorphism and human health.

## Conclusions

This study showed that the dietary intervention prevented an age-related increase in triglyceride levels in individuals with IFG or new-onset type 2 diabetes who possess the TT allele, but not the CT or CC allele, of the *APOA5* -1131 T > C polymorphism.

## Abbreviations

apo A-V: Apolipoprotein A-V; IFG: Impaired fasting glucose; SNP: Single-nucleotide polymorphism; CVD: Cardiovascular disease; HDL: High-density lipoprotein; LDL: Low-density lipoprotein; BMI: Body mass index; WHR: Waist hip ratio; BP: Blood pressure; HOMA-IR: Homeostasis model assessment of insulin resistance; hs-CRP: High-sensitivity C-reactive protein; MDA: Malondialdehyde; ba-PWV: Brachial-ankle pulse wave velocity; PUFA: Polyunsaturated fatty acids; SFA: Saturated fatty acids.

## Competing interests

The authors declare they have no competing interests.

## Authors’ contributions

Conceived and designed the experiments: MK, JSC, MK, S-HL, and JHL. Performed the experiments: MK, JSC, and MK. Analyzed the data: MK and MK. Contributed reagents/materials/analysis tools: S-HL and JHL. Wrote the paper: JHL and MK. All authors read and approved the final manuscript.
